# Investigating strategies used by hospital pharmacists to effectively communicate with patients during medication counselling

**DOI:** 10.1111/hex.12558

**Published:** 2017-03-30

**Authors:** Bernadette A. M. Chevalier, Bernadette M. Watson, Michael A. Barras, William Neil Cottrell

**Affiliations:** ^1^ School of Pharmacy The University of Queensland Brisbane QLD Australia; ^2^ Department of English The Hong Kong Polytechnic University Hung Hom Kowloon Hong Kong; ^3^ Pharmacy Department Princess Alexandra Hospital Brisbane QLD Australia

**Keywords:** Communication Accommodation Theory (CAT), communication, hospital pharmacist, patient

## Abstract

**Background:**

Medication counselling opportunities are key times for pharmacists and patients to discuss medications and patients’ concerns about their therapy. Communication Accommodation Theory (CAT) describes behavioural, motivational and emotional processes underlying communication exchanges. Five CAT strategies (approximation, interpretability, discourse management, emotional expression and interpersonal control) permit identification of effective communication.

**Objective:**

To invoke CAT to investigate communication strategies used by hospital pharmacists during patient medication counselling.

**Design:**

This was a theory‐based, qualitative study using transcribed audiorecordings of patients and hospital pharmacists engaged in medication counselling.

**Setting and participants:**

Recruited pharmacists practised in inpatient or outpatient settings. Eligible patients within participating pharmacists’ practice sites were prescribed at least three medications to manage chronic disease(s).

**Main outcome measures:**

The extent to which pharmacists accommodate, or not, to patients’ conversational needs based on accommodative behaviour described within CAT strategies.

**Results:**

Twelve pharmacists engaged four patients (48 total interactions). Exemplars provided robust examples of pharmacists effectively accommodating or meeting patients’ conversational needs. Non‐accommodation mainly occurred when pharmacists spoke too quickly, used terms not understood by patients and did not include patients in the agenda‐setting phase. Multiple strategy use resulted in communication patterns such as “information‐reassurance‐rationale” sandwiches.

**Discussion and conclusions:**

Most pharmacists effectively employed all five CAT strategies to engage patients in discussions. Pharmacists’ communication could be improved at the initial agenda‐setting phase by asking open‐ended questions to invite patients’ input and allow patients to identify any medication‐related concerns or issues.

## 1 INTRODUCTION

Effective communication skills are necessary for the provision of high‐quality patient care by health‐care professionals.^1^ Poor communication exchanges with patients have been associated with lower patient satisfaction, treatment non‐adherence and negative clinical outcomes.^2^, ^3^, ^4^ Communication takes place between hospital pharmacists and patients throughout the hospital stay to support patients in effectively managing their medications. Pharmacists do so by addressing patients’ medication concerns, discussing changes made to medication therapy, and by providing patients with medication information and education.^5^, ^6^, ^7^, ^8^ Hospital pharmacy practice has expanded in a number of countries to include advanced clinical skills such as prescriptive authority, ability to requisition laboratory tests, conduct physical assessments and provide immunizations.^9^, ^10^, ^11^, ^12^, ^13^ In addition to possessing competent clinical skills, pharmacists must further develop their ability to communicate effectively with other health‐care professionals and especially with patients and their caregivers.^14^, ^15^, ^16^, ^17^, ^18^, ^19^

Little has been published about the communication taking place between hospital pharmacists and patients with few details about what makes this exchange effective.^16^, ^17^, ^18^, ^19^, ^20^, ^21^ Most papers’ methodologies lack a theoretical basis or focused mainly on skill assessment.^17^, ^18^, ^19^, ^20^, ^21^, ^22^ Effective communication between pharmacists and patients may lead to improved clinical outcomes as described in the physician‐patient communication literature.^4^, ^23^ It is likely that effective exchanges between pharmacists and patients would better enable patients to make informed decisions about their medications.^23^ However, before relationships between effective pharmacist‐patient communication and patient outcomes can be explored, it is essential to first understand what is taking place in these communication exchanges.

This research is theory‐based and addresses these gaps in the hospital pharmacist‐patient communication literature by exploring the effectiveness of communication taking place between pharmacists and patients during medication counselling sessions. Medication counselling and patient medication counselling are frequently used terms to describe the pharmacist‐patient interaction where patients receive and exchange information about their medications.^21^, ^24^ Communication Accommodation Theory (CAT), widely used in health‐care communication research, will be the theoretical framework applied.^25^, ^26^, ^27^, ^28^, ^29^, ^30^ CAT has been used to interpret communication exchanges between parents and nurses in neonatal intensive care units,^25^ doctors and patients at end of life care,^30^ and also communication between hospital physicians of different specialties.^28^ CAT proposes that an individual's goals drive their communication behaviour.^31^ The person invokes CAT strategies to accomplish their goals and these are observed as communication behaviours.

There are five strategies described within CAT (approximation, interpretability, discourse management, emotional expression and interpersonal control) that will permit detailed analyses of hospital pharmacist‐patient interactions and the identification of aspects of effective communication.^25^, ^32^, ^33^ When speakers appropriately adopt these strategies, studies have shown that interactants rate the communication as effective.^34^ CAT research typically dichotomizes outcomes as being either accommodative or non‐accommodative. Accommodative behaviours reduce communication barriers between those interacting whereas non‐accommodation behaviours tend to create linguistic distances between speakers.^31^, ^33^

Approximation concerns how individuals adjust their speech patterns such as the pitch, rate, volume, tone, use of dialect or accents to converge towards or diverge from their partner's speech.^35^, ^36^ Appropriate approximation occurs when speakers perceive that their speech patterns complement each other. Interpretability strategies focus on each speaker's conversational competence. Interactants who modulate their language and word choice to ensure their words are understood demonstrate appropriate interpretability. While interpretability highlights conversation content, discourse management strategies involve conversation processes to promote conversation between interactants. Appropriate discourse management strategies accomplish this through turn‐taking, changing topics as needed, responding to non‐verbal cues and using conversational repair such as face maintenance that allows patients to maintain a positive self‐image and prevents interactions from becoming ineffective or negative.^25^, ^36^ Accommodative emotional expression takes place when pharmacists provide appropriate levels of reassurance and empathy in response to patients’ emotional needs.^32^ Interpersonal control focuses on the roles and power relations between speakers. Appropriate interpersonal control strategies used by pharmacists promote equality between themselves and patients, not constraining patients to passive patient roles.^25^ However, power imbalances between patients and pharmacists will always be inherent, because pharmacists hold specialized knowledge and information not always accessable to patients. These relationships between health‐care providers and patients have been described by researchers in health‐care communication as intergroup.^25^, ^37^ Specifically, pharmacists self‐identify as members of a “health‐care professional” group while those they counsel belong to the “patient” group. CAT proposes that although health‐care professionals may move between these intergroup identities (social and professional) and personal identities (individual likes and dislikes), it is often their intergroup identities that are most salient.^38^

Invoking CAT provides a vehicle for interpreting the detailed patterns and flow of pharmacist‐patient conversations and will help identify occasions of accommodation or non‐accommodation. These may highlight areas of strength as well as areas in which pharmacists’ communication skills require further development to improve the effectiveness of their exchanges with patients.

This research represents a portion of a PhD project that will include perspectives of pharmacists and patients about their shared medication counselling experience. However, this part of the study will focus on the observed pharmacist‐patient interactions only. The aim of this study is to invoke CAT to investigate communication strategies used by hospital pharmacists during patient medication counselling.

## 2 METHODS

Qualitative methods were used to gather in‐depth communication exchanges between pharmacists and patients during medication counselling. Research ethics approval was received from Human Research Ethics Committees of the participating hospital and university (HREC/15/QRBW/433). Refer to the Appendix for a completed Consolidated Criterion for Reporting Qualitative Studies (COREQ) document.^39^

### 2.1 Recruitment and data collection

This study took place at a large 1000 bed tertiary teaching hospital with multiple specialties including inpatient wards and outpatient clinics.

#### 2.1.1 Inclusion criteria

Pharmacists: Interested hospital pharmacists whose current professional duties included the provision of clinical pharmacy services in an inpatient and/or outpatient setting.

Patients: Interested patients admitted to either an inpatient or outpatient setting in which clinical pharmacy services were provided by a pharmacist participating in this study; Patients who have been prescribed three or more medications to manage a chronic disease

#### 2.1.2 Recruitment

Pharmacists were recruited first. An electronic invitation was sent to approximately 50 departmental pharmacists. Eight pharmacists expressed interest initially, and the remaining four pharmacists responded following a reminder email sent 3 weeks later. Pharmacists’ demographics were examined and compared to those from a national demographic survey to ensure an accurate representation.^40^ Because the pharmacists’ ages, qualifications, experience and practice site reflected demographics from within the hospital and nationally, further purposive sampling was not required.

A convenience sample of interested patients meeting the inclusion criteria were first identified by their nurse and then approached by BC who provided study details, obtained consent and gathered patient demographic data.

#### 2.1.3 Data collection

Participating hospital pharmacists and patients were audiorecorded while engaged in medication counselling. Each pharmacist counselled four different patients to observe how they used and adjusted their communication skills to meet the conversational needs of unique individuals. BC observed the pharmacist‐patient interactions in person and out of the direct view of the participants to record non‐verbal communication taking place between the participants and to make notes about environmental conditions that might have affected the quality of their exchange. Prior to conducting audiorecorded medication counselling sessions, BC accompanied pharmacists interacting with patients on their ward. This was an attempt to normalize BC's presence and minimize its effect on pharmacists’ performances during audiorecorded sessions. As well, pharmacists were told that the study's aim was to investigate whether effective communication takes place between hospital pharmacists and patients; however, pharmacists were intentionally not informed about CAT behaviours to be observed during medication counselling.

### 2.2 Data analysis and coding

Both patient and pharmacist demographic information and questionnaire results were descriptively analysed. The actual names of pharmacists were not used, but were replaced by pseudonyms that appear in exemplars provided.

Audio recordings were transcribed verbatim and verified by comparing transcripts with original audiorecordings to reconcile any discrepancies, wherever possible. Transcripts were selectively coded for the five CAT strategies in pattern‐based discourse analysis as described in the literature.^41^

Samples of selectively coded transcripts and their corresponding audiorecordings were checked by co‐researcher (BW) to ensure appropriate and consistent interpretation. Transcripts were coded twice, initially grouped according to study pharmacist and then rechecked and recoded, if necessary, in the order of patient enrolment into the study. Audiorecordings were referenced to verify correct interpretation of tone and intent of dialogue. NVivo^®^ software was used to assist in code organization. Observational field notes were collected during audiorecorded medication counselling sessions and reflexive note taking occurred throughout the study.

### 2.3 Reflexivity

The main researcher (BC) conducted reflexive note taking throughout the study. BC is a Caucasian female with more than 20 years’ experience as a hospital pharmacist in Canada. She holds strong opinions about best practice and professionalism and is aware of how these could possibly influence her perception and interpretation of the data as a researcher. As well, being aware of BC's experience as a hospital pharmacist may have made pharmacists more conscious or nervous having a colleague observe their practice, and some may have felt that their skills were being judged or critiqued. BC had not previously worked with any of the participating pharmacists.

## 3 RESULTS

### 3.1 Medication counselling sessions

Twelve pharmacists engaged four patients each for a total of 48 medication counselling interactions that took place between November 2015 and April 2016. Initially, the intent was to recruit 15 pharmacists (each engaging four patients) for a target of 60 pharmacist‐patient interactions. However, by 40 pharmacist‐patient exchanges no new applications of the five CAT strategies were observed. A decision was made, by the research team, to have the 12 pharmacists complete all four patient interactions for a final number of 48 pharmacist‐patient conversations.

Almost all the inpatient pharmacist‐patient exchanges took place at the patient's bedside, within busy, noisy four bed bays. Only a few inpatient conversations occurred in private rooms or in quiet patient lounges. Medication counselling in outpatient settings took place in both private clinic rooms and office areas within open spaces depending on availability.

The time to complete patient counselling sessions varied considerably, depending on needs of the patient and complexity of drug regimens with a mean time of 13.6 minutes (range from 3.8 to 45.2 minutes).

### 3.2 Participant characteristics

Study pharmacists’ demographic characteristics are shown in Table 1. These are fairly consistent with demographics reported for Australia except more study pharmacists had postgraduate training (58% vs 26%).^40^ Table 2 includes participating patient demographic characteristics. Compared to overall hospital age and gender demographics obtained for the study period, the study patients were older (average age of 63.1 vs 52 years of age) and included fewer female study patients (44% vs 53%).

**Table 1:** Pharmacist demographics

Demographic characteristics (n=12)	Number (%)
Female gender	10 (83)
Age range	
21‐30	6 (50)
31‐50	5 (42)
>50	1 (8)
Highest level education (Pharm)	
B Pharm	4 (33)
B Pharm (Hon)	1 (8)
Graduate Diploma (Clinical Pharm)	3 (25)
Masters (Clinical Pharm)	4 (33)
Pharmacist experience as pharmacist (years)	
1‐5	5 (42)
6‐10	1 (8)
11‐15	3 (25)
16‐20	2 (17)
>21	1 (8)
Clinical practice area	
Inpatient (General medicine, Cardiology, Oncology, Nephrology, Neurology, Geriatrics, Surgery and Emergency)	9 (75)
Outpatient (Infectious diseases clinic, Heart failure clinic and Renal clinic)	3 (25)

**Table 2:** Patient demographics

Demographic characteristics (n=48)	Number (%)
Female gender	21 (44)
Age range	
21‐40	2 (4)
41‐50	9 (19)
51‐60	10 (21)
61‐70	12 (25)
71‐80	10 (21)
>80	5 (10)
Average age	63.1
Patient care area	
Inpatient	36 (75)
Cardiology	6
Emergency	4
Geriatrics	3
General medicine	5
Nephrology	2
Neurology	2
Oncology	2
Surgery	12
Outpatient	12 (25)
Heart failure clinic	4
Infectious diseases clinic	3
Renal clinic	5
Number of medications	
Mean number of medications/patient	11.1
Standard deviation	±5.3
Range in numbers of medications/patient	4‐30

### 3.3 Discourse analyses—Hospital pharmacists’ utilization of CAT strategies

The following analyses examines how pharmacists accommodate, or not, to patients’ conversational needs during medication counselling based on the five CAT strategies.

#### 3.3.1 Approximation

Study pharmacists demonstrated accommodative approximation behaviours by matching patients’ speech patterns (volume, pace and accent). Pharmacists occasionally used colloquialisms when speaking to patients, likely to put patients at ease and to encourage engagement in the conversation. Phrases used were often greetings such as “G'day again…” or when conversations were winding down, for example “That's alright mate. All the best hey.” Some pharmacists used expressions such as “Righto” when breaks occurred in the conversation. One pharmacist appropriately accommodated a patient by matching their accent after establishing a shared UK origin.

With a few exceptions, pharmacists spoke clearly and with adequate volume and pace to ensure patient understanding. This was based on observing patients’ nod or making utterances to indicate they were following pharmacists’ explanation. However, a few pharmacists spoke too quickly, potentially not accommodating patients’ conversational needs. In rare occasions, underaccommodated patients were observed to respond to the excessive speech rate by disconnecting or engaging less with pharmacists. Interestingly, these pharmacists were aware of their behaviour but identified different reasons for doing so. One pharmacist attributed her rapid speech to her highly energetic nature, whereas two others remarked that time constraints and pressure to discharge patients necessitated speaking quickly as it served to prevent conversations from straying off topic.

Sometimes pharmacists varied their speech rate depending on the focus of conversation. They used a faster pace when discussing interpersonal issues, but a slightly slower, more thoughtful and pronounced pace when emphasizing key points in therapy such as side‐effect management.

#### 3.3.2 Interpretability

Most pharmacists adopted appropriate interpretability strategies using easy‐to‐understand phrasing, explained rationale for medication therapy with fairly simple terms and effectively worded questions to elicit patient responses. Patient‐specific medication lists were used by all pharmacists as visual guides to support their verbal explanations and often arranged to have medications at hand to emphasize their identity or demonstrate their use. Many pharmacists also used hand gestures or drew cartoon diagrams to indicate how medications work in the body.

In the following example, the pharmacist displayed appropriate interpretability using everyday language to describe how a patient might experience muscle weakness and soreness associated with certain cholesterol lowering agents:

“…the side effect to watch out for is unusual muscle pains or aches. The way that I've heard patients describe it before is… ‘My legs and my arms‐ they're weak… they're aching‐ it's not going away.’”  (Karen)

At times, pharmacists used medical jargon and terminology, but provided an explanation immediately afterwards, for example: “… in terms of analgesia or your pain relief …”

Occasionally pharmacists demonstrated inappropriate interpretability strategies by using terminology that was not understood by patients who responded with quizzical facial expressions or directly asked for the term's meaning. In this example, the pharmacist used the term “neuralgia” as opposed to “nerve pain.”

“So this is the one that the pain team have suggested…with the kind of neuralgia that you've got.” (Christine)

Pharmacists typically adjusted the terminology they used in their interactions to match that understood by the patient. In this exchange, an inpatient pharmacist intentionally and appropriately used medical terms to describe the patient's heart condition to a patient, who was also a retired nurse.

“…the next one…Coversyl or Perindopril. That one… it is used for high blood pressure but it's also just used after cardiac events, to protect your cardiovascular system…” (Ingrid)

#### 3.3.3 Discourse management

Discourse management strategies utilized by study pharmacists to engage patients in conversations included asking open‐ended questions, promoting two‐way conversations by turn‐taking, demonstrating careful listening, paying attention to non‐verbal cues and using conversational repair such as face maintenance or conversation maintenance such as back‐channelling (eg “hmm” and “yeah”).

Many pharmacists engaged patients by asking open‐ended questions. An outpatient pharmacist inquired about how a diabetic patient managed hypoglycaemia symptoms, “What do you do… when you feel like that?”

However, not all study pharmacists consistently used open‐ended questions in discussions with patients about their medications. Instead, some pharmacists avoided open‐ended questions and only asked patients a few questions near the end of the patient counselling session such as “Do you have any questions about any of that…?”

Pharmacists employed a number of techniques to encourage patient interaction or elicit a response often using carefully constructed questions or phrases such as “What's your understanding of what you're on that for?” or “How are you going with the metoprolol? Remind me what dose that one is.”

Many pharmacists also engaged patients by incorporating pauses at different points throughout the delivery of medication information with phrases such as “alright?” or “okay?”. This not only verified patients’ understanding, but also gave patients an opportunity to interject or ask questions about the information.

Sometimes pharmacists used conversation repair strategies such as face maintenance as demonstrated in the dialogue below by Ellen, an inpatient pharmacist, who down‐played a mistake made by a patient to allow him to “save face.”

Patient That is supposed to be two lots, not one! I was supposed to get two lots of 20 mL, once in the morning, two lots of 20 mL, twice a day, for the Kapanol.

Pharmacist Oh.

Patient For the slow release. See, yeah it's got one. Oh sorry…

Pharmacist One capsule, twice a day.

Patient …no, no, twice a day. Sorry … no, sorry. Because I saw it… sorry, no, no‐ it's me.

Pharmacist No, that's okay… No, they can be a little bit confusing to read…

All pharmacists included non‐verbal gestures to encourage patients’ engagement in their conversations. Examples included eye contact, nodding, facial expressions, hand gestures, leaning in towards patients when speaking or while listening carefully, and “back‐channelling” (ie uh‐huh, hmm).

#### 3.3.4 Emotional expression

Accommodative emotional expression took place when study pharmacists recognized and demonstrated an appropriate level of empathy and reassurance in response to patients’ emotional needs. Pharmacists accomplished this by using both verbal and non‐verbal communication to express kindness and humour and validate patients’ concerns. However, there were rare occasions where pharmacists appeared impatient or even brusque with patients.

The way in which appropriate emotional expression was demonstrated depended on the individual pharmacist‐patient conversation, what was needed in the situation, and each pharmacist's interaction style based on their own preferences and experiences. Some pharmacists were tactical in their application of emotional expression. In this example, Fiona, an inpatient pharmacist, used a kindly approach when relaying large amounts of information to patients by breaking up these segments with reassurance creating an information‐reassurance‐information sandwich.

“The other thing with antibiotics…so this one doesn't cause too many side effects at all ‐ and you've been on it for a few days. Sometimes it can cause a bit of an upset stomach, but if you do get some severe diarrhoea with it then you certainly need to let your doctor know as well, okay?” (Fiona)

Study pharmacists were frequently observed recognizing patient cues and validating patients’ concerns using expressions such as “It can be a bit overwhelming…It can be a bit much to take in at once…”

In Australia, patients are required to contribute to the cost of medications prescribed for use in the community. Many pharmacists showed sensitivity to patients’ ability to afford medications by working with physicians to change patients’ medications to those covered under the national subsidized medicines programme or through other strategies, “… Do you know about the Safety Net…where you reach a certain limit and you get it free?”

#### 3.3.5 Interpersonal control

Numerous accommodative interpersonal control strategies were used by study pharmacists to promote equality between themselves and patients, and to empower or give patients agency, encouraging them to be active participants in their health care.

Study pharmacists varied in how they demonstrated and negotiated interpersonal control within their intergroup or professional role and within their interpersonal interactions. Some pharmacists assumed a professional role throughout their patient interactions while others seemed adept at switching roles from interpersonal to their pharmacist role, often by changing their voice to a more officious professional tone just before launching into a discussion about medications.

Inappropriate interpersonal control was observed particularly when medication counselling was initiated. Here, pharmacists affirmed their professional identities and intergroup relationship with patients by controlling the agenda‐setting phase. This control often continued throughout pharmacist‐patient interactions when pharmacists’ communication strategies ensured that conversations stayed on track. Almost all pharmacists began patient medication counselling by first introducing themselves, and then stating their reason for the conversation. Patients were rarely asked at the onset whether they had any specific medication matters or concerns they would like to discuss. This example typifies the approach used by most pharmacists:

“…So we'll just start with the list… it goes through your details, medicine name, brand name, uses, like reasons for taking them… We'll go through them. We'll go through them one at a time…” (Laura)

An interpersonal control strategy utilized by many pharmacists to ensure collection of relevant medication information was to redirect conversations that strayed into more social topics. Most pharmacists appropriately accomplished this by listening politely to these diversions before redirecting conversations by tactfully changing the topic using expressions such as “lovely” or “okay,” pausing and then prompting the patient about the previous discussion point. An outpatient pharmacist skilfully executed this with gentle tact, “That does it. Yeah. Lovely… So you've been using the insulin since the transplant.”

Although study pharmacists tended to lead and direct conversations, most patients appeared comfortable to interject to demonstrate their understanding or clarify information. Most pharmacists appeared non‐plussed by patients’ interruptions and addressed questions or queries as they arose reflecting appropriate interpersonal control. On rare occasions, pharmacists would show their impatience or annoyance by answering abruptly, increasing the language level using medical terms or speaking over the patient to bring the topic back on track. These are all examples of inappropriate interpersonal control communication behaviours as pharmacists exerted their professional role.

However, pharmacists displayed a number of behaviours to promote patient autonomy and participation in their medication management. One strategy used by pharmacists was to incorporate the rationale for treatment with information and advice provided. This delivery method resembled an information‐reassurance‐rationale sandwich where medication information was followed by reassurance and then explanations for any medication changes made. Here, information relates to “interpretability,” reassurance to “emotional expression” and “rationale” is the “interpersonal control” CAT strategy.

An example where rationale was provided to encourage self‐monitoring is as follows:

“If you're brushing your teeth and you're getting bleeding gums… nose bleed that you can't stop… all of those are signs that actually you've probably got too much in your system and you need to see a doctor.” (Christine)

Pharmacists positively reinforced patients’ correct understanding of their medications, effective medication management strategies, and encouraged patients’ autonomy in making appropriate health‐care decisions, as shown in this exemplar:

“You know yourself best. So you just keep an eye out and if you're experiencing any usual symptoms‐ pop off to the GP‐ it could be the medication.” (Ellen)

Involving patients in shared decision‐making and assisting patients to successfully navigate complex health‐care systems were other ways in which study pharmacists empowered patients. This exemplar illustrates how an outpatient pharmacist provided a patient assistance by connecting her to key health‐care professionals in her community in preparation for an upcoming bus tour.

“…what I will do is suggest to the doctors that we do give you one of those Glucagon pens and we'll get your pharmacy, your local pharmacy to show you how to use it just in case something happens. When you are on the tour make sure you let the tour guide know that you are a diabetic….” (Geena)

## 4 DISCUSSION

Investigating how hospital pharmacists used CAT strategies in their conversations with patients highlighted both strengths and weaknesses in their repertoire of communication skills. For the most part, pharmacists demonstrated accommodative approximation strategies (speech rate, tone, volume or accent). Non‐accommodative behaviour has been described by researchers when health‐care professionals caring for elderly patients oversimplified their speech with emphatic enunciations, unnecessarily loud tones and reduced speech rate.^42^ Pharmacists in this study were not observed to exaggerate their speech in this manner. Alterations in their tone and rate were subtle and made to emphasize key medication points.

In terms of interpretability, study pharmacists typically were conscientious about choosing language and terminology that was understood by patients. This is in contrast to findings from researchers who reported pharmacists frequently used medical terms not comprehended by patients during medication counselling.^16^, ^17^, ^22^, ^43^

Pharmacists adopted a number of accommodative discourse management strategies to engage patients in conversation. By interspersing questions such as “Okay?” or “Alright?” throughout the information provided, pharmacists gave patients an opportunity to interject or ask questions about the information as well as to check for patient understanding. Greenhill et al. depicted this activity as “chunk” and “check.” However, they found that their study pharmacists delivered “chunks” of information, but did not check for patients’ understanding.^22^ Researchers studying pharmacists counselling patients in South African HIV clinics termed this as “response solicitations” where pharmacists used these prompts to both invite patients to ask questions and check their understanding.^44^ Using open‐ended questions was another technique utilized by our study pharmacists and identified by other researchers as an effective method to elicit patient engagement and highlight any patient concerns and preferences.^45^, ^46^, ^47^ However, researchers studying hospital pharmacist communication with patients have found that pharmacists rarely use open‐ended questions to engage patients during medication counselling.^14^, ^22^, ^43^

Accommodative emotional expression (appropriate levels of empathy and reassurance) was nearly always demonstrated by study pharmacists. Validating patients’ concerns is an important process for pharmacists to assist patients in dealing with negative emotions and worries about their medications and health care.^48^ Sensitivity to patients’ ability to afford their medications was another example of caring behaviour demonstrated by many study pharmacists. This contrasted with other Australian research that found hospital pharmacists did not explore financial reasons for patients’ medication issues.^43^

There were rare occasions where participating pharmacists appeared impatient or even brusque with patients, not unlike descriptions included in the literature.^22^, ^43^, ^49^ Perhaps, this was related to pharmacists’ perceived time pressures to stay on task. Effective demonstration of empathy and compassion by health‐care professionals has been reported to result in greater patient satisfaction^2^, ^50^, ^51^ and adherence to therapy.^4^

Numerous appropriate interpersonal control strategies were used by pharmacists to give patients agency and encourage patients to take an active role in their health care. Research supports health‐care professionals empowering patients to effectively self‐manage chronic conditions.^52^, ^53^, ^54^ Study pharmacists frequently discussed the medication's rationale when counselling patients about their medications. This practice has been previously reported by pharmacists to help patients understand reasons for therapy, promote adherence and encourage patient participation in their health care.^55^

However, not all interpersonal control strategies adopted by pharmacists were accommodative. Pharmacists rarely encouraged patient input at the start of their conversations, the agenda‐setting phase. Patient collaboration should be encouraged from the onset using open‐ended questions such as “What sorts of medication concerns or questions would you like to talk about?” This is in contrast to the current situation where pharmacists inform patients how the conversation will unfold and then continue onward in a task‐oriented approach. Including patients in agenda‐setting by eliciting their expectations for the conversation has been favourably described in physician research.^56^, ^57^, ^58^, ^59^ Not engaging patients in the design and purpose of the medication counselling session could be interpreted as under‐accommodating patients’ power and relationship needs by not allowing their input into this initial stage.

Another demonstration of inappropriate control observed was related to pharmacists’ task‐oriented approach to medication counselling. All pharmacists used patient‐specific medication lists that acted as visual props and information sources for patients, but also as a mechanism to keep conversations on track. The behaviour of task‐oriented pharmacists could be considered non‐accommodative if they responded inappropriately to patients’ questions or interruptions by showing annoyance or talking over them. On the other hand, pharmacists who respond respectfully and address patients’ queries as they arise are demonstrating appropriate interpersonal control. Using a methodical approach in medication counselling reflects a well‐entrenched process learned in most undergraduate pharmacy programmes and assumed throughout clinical practice.^60^, ^61^, ^62^, ^63^, ^64^ Similar agenda‐setting and task‐oriented pharmacist behaviours were reported by other researchers.^21^, ^22^, ^43^, ^44^

Most pharmacists effectively applied CAT strategies as they adapted to patients’ conversational needs, often combining different conversational techniques (eg information‐reassurance‐rationale sandwich). This was likely done to prevent patients from feeling overwhelmed and to ensure patients felt confident with medication information provided. Pharmacists’ use of multiple CAT strategies is not surprising as it likely reflects their numerous goals in communicating with patients.

A number of potential study limitations exist. Having pharmacist‐patient conversations observed and recorded may have resulted in expected or professional desirable behaviour by pharmacists and may not be representative of usual practice (Hawthorne effect).^65^ Attempts were made to mitigate this effect and normalize medication counselling by having BC shadow pharmacists interacting with patients prior to the recorded interview. As well, patients may have assumed socially desirable behaviour knowing their interaction was being observed and recorded. Another potential limitation was the self‐selection of motivated pharmacists enrolling in this study which may limit transferability of positive results. Because this study was conducted at a single public hospital, the results might not be transferable to all specialty areas at other sites, rural or private hospitals. Although participant groups were relatively heterogenous (Tables 1 and 2), specialized clinical areas such as those with mental health patients or persons with dementia were not represented.

This study demonstrates how CAT can be successfully applied to pharmacist‐patient conversations to obtain detailed interpretations and insight into the communication behaviours taking place. Previous research invoking CAT to examine physician and nurse communication with patients shares similar findings including patient identified preferences such as empathy (emotional expression) and receiving well explained information in layman's terms (interpretability).^25^, ^29^, ^30^, ^66^ However, to our knowledge, this study is novel in its application of CAT to investigate pharmacist‐patient interactions.

These findings will inform both pharmacy students’ and practitioners’ communication skills training and assessment to enable their effective integration of CAT communication strategies in conversations with patients. Patients who engage in effective communication exchanges with pharmacists may be better able to manage their medications, experience more satisfaction with their interaction, which in turn may lead to improved medication taking behaviours. Future research observing CAT behaviours in pharmacists should be expanded to include exchanges between pharmacists and other health‐care professionals as well as with patients in different health‐care settings such as primary care or community pharmacies.

## 5 CONCLUSIONS

Most pharmacists effectively employed all five CAT communication strategies during patient counselling sessions as they adapted to patients’ conversational needs. However, individual methods of discourse with patients varied considerably and were likely related to pharmacists’ preferred communication strategies. Pharmacists’ communication could be improved at the initial agenda‐setting phase by asking open‐ended questions to invite patients’ input and empower patients to raise any concerns or issues they might have about their medications.

## CONFLICTS OF INTEREST

There are no conflicts of interest to disclose.

## APPENDIX 1

### Consolidated criteria for reporting qualitative studies (COREQ)

**Table:**

No. item	Guide question/description	Reported on Page #
**Domain 1: Research team and reflexivity**		
Personal characteristics		
1. Interviewer/facilitator	Which author(s) conducted the interview or focus group?	Page 3
		No interviews or focus groups held for this part of the PhD research; BC audio recorded, observed, and took notes during pharmacist‐patient medication counselling sessions
2. Credentials	What were the researcher's credentials?	Title Page
		BC (PhD Candidate)
3. Occupation	What was their occupation at the time of the study?	Title Page
4. Gender	Was the researcher male or female?	Female
5. Experience and training	What experience or training did the researcher have?	Experience conducting and analysing data from focus groups of pharmacists; Formal training through specialised qualitative methods courses and instruction from experienced advisors/co‐researchers.
Relationship with participants		
6. Relationship established	Was a relationship established prior to study commencement?	BC had met many of the pharmacists who had been involved in earlier focus groups, but has never worked as a pharmacist with any of the pharmacists; BC did not know any of the patients prior to the study
7. Participant knowledge of interview	What did the participants know about the researcher? E.g. personal goals, reasons for doing research	All participants knew that the researcher was an experienced hospital pharmacist; participants were aware that the study was part of researcher's PhD project
8. Interviewer characteristics	What characteristics were reported about the interviewer/facilitator? E.g. bias, assumptions, reasons and interests in research topic	Page 3
		Discussed in the sub‐heading Reflexivity within the Methods section.
**Domain 2: Study design**		
Theoretical framework		
9. Methodological orientation and theory	What methodological orientation was stated to underpin the study?	Pages 2
		Communication Accommodation Theory (CAT)
Participant selection		
10. Sampling	How were the participants selected? E.g. purposive, convenience, consecutive, snowball	Page 3
		All pharmacists were invited to take part. Then demographics verified to reflect that of department and national hospital pharmacists. (Convenience and purposive); Convenience sample of patient participants admitted to pharmacists’ practice area and meeting criteria
11. Method of approach	How were participants selected? E.g. face‐to‐face, telephone, mail, email	Page 3
		Pharmacists—email; Patients—face‐to‐face
12. Sample size	How many participants were in the study?	Page 3‐4
13. Non‐participation	How many people refused to participate or dropped out? Reasons?	Pharmacists—all 12 pharmacists who consented completed the study; Patients—all 48 patients who consented completed the study
Setting		
14. Setting of data collection	Where was the data collected? E.g. home, clinic, workplace	Page 3
		All pharmacist‐patient medication counselling sessions took place within the hospital either on inpatient wards or within outpatient clinics
15. Presence of non‐participants	Was anyone else present besides the participants and researchers?	Yes, most inpatient interactions took place at the patient's bedside, therefore other patients, their families and other healthcare professionals were nearby; outpatient conversations took place in both private clinic rooms as well as shared open areas with other patients and healthcare professionals present
16. Description of sample	What are the important characteristics of the sample? E.g. demographic data, date	Page 4‐5 (Tables 1 and 2) Pharmacist and patient demographic tables include this data. Data collection occurred from November 2015‐April 2016.
Data collection		
17. Interview guide	Were questions, prompts, guides provided by the authors? Was it pilot‐ tested?	Not for this study.
18. Repeat interviews	Were repeat interviews carried out? If yes, how many?	N/A
19. Audio/visual recording	Did the research use audio or visual recording to collect the data?	Page 3
		All pharmacist‐patient interactions were audio recorded by BC.
20. Field notes	Were field notes made during/or after the interview or focus group?	Page 3
		Field notes were taken during pharmacist‐patient interactions, and reviewed at time of analysis.
21. Duration	What was the duration of the interviews or focus groups?	Page 4
		Patient counselling sessions took an average of 13.6 minutes to complete (range 3.8‐45.2 min)
22. Data saturation	Was data saturation discussed?	Page 3‐4
		Saturation of data determined after 40 medication counselling sessions (no new applications of the five CAT strategies observed.)
23. Transcripts returned	Were transcripts returned to participants for comment and/or correction?	No
**Domain 3: Analysis and findings**		
Data analysis		
24. Number of data coders	How many data coders coded the data?	Page 3
		Mainly BC; however, coding samples with audio recordings were verified by co‐researcher/advisor BW
25. Description of the coding tree	Did the authors provide a description of the coding tree?	No. Selective coding was conducted and based on the five CAT strategies.
26. Derivation of themes	Were themes identified in advance or derived from the data?	Page 3
		Themes were identified in advance (ie data was selectively coded)
27. Software	What software, if applicable, was used to manage the data?	Coding was done manually; NVivo 11 used to help organise the codes
28. Participant checking	Did participants provide feedback on the findings?	No
Reporting		
29. Quotations presented	Were participant quotations presented to illustrate themes/finding? Was each quotation identified? E.g. participant number	Pages 4‐7
		Quotations from pharmacists were identified by pseudonyms (actual names were not used).
30. Data and finding consistent	Was there consistency between the data presented and the findings?	Yes
31. Clarity of major themes	Were major themes clearly presented in the findings?	Pages 4‐7
32. Clarity of minor themes	Is there a description of diverse cases or discussion of minor themes?	Pages 4‐7
		Data was themed by the five CAT strategies; findings and discussion included description and examples of both accommodative and non‐accommodative behaviours
